# High-speed X-ray imaging pixel array detector for synchrotron bunch isolation

**DOI:** 10.1107/S1600577515022754

**Published:** 2016-01-28

**Authors:** Hugh T. Philipp, Mark W. Tate, Prafull Purohit, Katherine S. Shanks, Joel T. Weiss, Sol M. Gruner

**Affiliations:** a Laboratory of Atomic and Solid State Physics, Cornell University, Ithaca, NY 14853, USA; b Cornell High-Energy Synchrotron Source (CHESS), Cornell University, Ithaca, NY 14853, USA

**Keywords:** imaging detector, high frame rate, X-ray detector, pixel array detectors, time-resolved imaging, area detector

## Abstract

A wide-dynamic-range imaging X-ray detector designed for recording successive frames at rates up to 10 MHz is described. X-ray imaging with frame rates of up to 6.5 MHz have been experimentally verified. The pixel design allows for up to 8–12 frames to be stored internally at high speed before readout, which occurs at a 1 kHz frame rate. An additional mode of operation allows the integration capacitors to be re-addressed repeatedly before readout which can enhance the signal-to-noise ratio of cyclical processes. This detector, along with modern storage ring sources which provide short (10–100 ps) and intense X-ray pulses at megahertz rates, opens new avenues for the study of rapid structural changes in materials. The detector consists of hybridized modules, each of which is comprised of a 500 µm-thick silicon X-ray sensor solder bump-bonded, pixel by pixel, to an application-specific integrated circuit. The format of each module is 128 × 128 pixels with a pixel pitch of 150 µm. In the prototype detector described here, the three-side buttable modules are tiled in a 3 × 2 array with a full format of 256 × 384 pixels. The characteristics, operation, testing and application of the detector are detailed.

## Abstract 2

A high-speed pixel array detector for time-resolved X-ray imaging at synchrotrons has been developed. The ability to isolate single synchrotron bunches makes it ideal for time-resolved dynamical studies.

## 1. Introduction   

The pulsed nature of X-ray synchrotron radiation sources may be exploited for time-resolved studies. Typical storage ring beam currents are in the range 100–500 mA, distributed among bunches that are tens of picoseconds in duration. The charge in each bunch and the inter-bunch spacing may vary with the mode of operation of the storage ring, with different bunch structures used to achieve different experimental goals. As examples, the 16 bunch mode at the European Synchrotron Radiation Facility (ESRF) consists of 48 ps duration bunches every 176 ns, whereas the Advanced Photon Source (APS) uses 24 bunches of 33.5 ps duration every 153 ns in one of its standard modes.

The ability to isolate single synchrotron X-ray pulses allows for dynamic studies limited in temporal resolution by the X-ray pulse duration. Mechanical chopper systems have been designed to transmit one X-ray pulse at a duty cycle of around a few kHz (Cammarata *et al.*, 2009 ▸). When synchronized, for example, to a laser in a pump–probe experiment, choppers allow use of conventional and relatively slow X-ray detectors, such as CCDs (Cho *et al.*, 2010 ▸), for ultrafast experiments. The interval between the excitation laser pump and the X-ray probe can then be progressively advanced in repeated experiments to map out the temporal response of the sample. This type of experiment must be repeated over many successive pump–probe cycles, requiring the sample to behave repeatably.

However, many dynamic problems of interest may differ in detail from sample to sample and/or can only be performed at relatively low repetition rates. Examples include crack propagation, materials failure under compressive impact, turbulence and response to intense magnetic pulses. In these cases, an imaging detector capable of acquiring multiple frames in rapid succession to create X-ray movies of dynamic processes provides direct observation of potentially unique dynamics. It may be desirable, in some cases, to monitor the response of the sample at uniformly spaced time intervals after the excitation. In other cases, it may be desirable to monitor with intervals spaced logarithmically in time. Often, the ideal sequence of durations between successive observations is not obvious until an experimental run starts. In such instances, it is important that the detector has flexibility to allow on-line rapid reprogramming of the intervals between each frame.

Other desirable characteristics of a fast-framing detector include a wide dynamic range per pixel per frame (*i.e.* ranging from single X-ray sensitivity to >10^3^ X-rays per pixel per frame), a linear response to integrated X-ray dose, a low rate of accumulated radiation damage, and a fast frame readout after each experiment. This article describes an X-ray imaging detector designed to have these characteristics.

The pulsed nature of the X-ray source precludes the use of X-ray photon-counting detectors in many of the above-mentioned applications (Campbell, 2011 ▸; Kraft *et al.*, 2009 ▸). X-ray photon-counting detectors process pulses from individual X-rays and increment an in-pixel digital counter. Multiple X-rays arriving in a single 40 ps synchrotron pulse, for example, cannot be resolved by counting circuitry. In order to see just two X-ray photons per pulse, a counting detector would need to be at least three orders of magnitude faster than presently available speeds. Fortunately, the total charge generated in a short X-ray pulse is proportional to the number of incident X-rays and can be measured with in-pixel charge-integrating circuitry (Graafsma *et al.*, 2015 ▸; Gruner *et al.*, 2001 ▸). In such an integrating detector, the X-rays that are stopped in a pixelated X-ray sensor layer, such as silicon, produce a number of electron–hole pairs proportional to the total energy of the stopped X-rays. If the thickness of the silicon is fully depleted by an applied bias, charges are separated and are collected at the input node of an integrating amplifier in each pixel. The integrating amplifier converts the charge to a voltage that may be sampled (and stored) on a capacitor within the pixel. Successive frames can be stored rapidly by sampling the integrator voltage using an array of capacitors within a pixel, resetting the amplifier to zero before the beginning of each new frame. The minimum frame time is set by the slew rate of the amplifier and the settling time required for a given accuracy. After the desired number of frames is stored on the capacitor array, the array is read by sequencing the stored analog voltages to an analog-to-digital converter.

We previously developed an early version of this technology (Barna *et al.*, 1997 ▸; Rossi *et al.*, 1999 ▸) capable of recording up to eight frames at microsecond rates. This pixel array detector (PAD), the first PAD (of either the integrating or photon-counting variety) to be used for hard X-ray synchrotron science applications, has been used to study shock waves (MacPhee *et al.*, 2002 ▸), fuel injection cycles (Liu *et al.*, 2009 ▸) and the phase behavior of exothermic reactive foils (Trenkle *et al.*, 2010 ▸). Although this detector has produced much science, it utilizes outdated technology and suffers from radiation damage and other operational limitations. The detector described in this article is a second-generation device that overcomes some of the limitations. The design of the pixel was tested in a 16 × 16 pixel format (Koerner, 2010 ▸; Koerner *et al.*, 2009 ▸; Koerner & Gruner, 2011 ▸). The detector described in this paper is a full-scale application-specific integrated circuit (ASIC) suitable for X-ray imaging experiments at synchrotrons. The ASIC is three-side buttable with wire-bonds along one edge, allowing for efficient tiling to cover a large area with multiple ASICs. The detector is called the Keck-PAD, as its development was largely funded by a generous award from the W. M. Keck Foundation.

The advent of X-ray free-electron lasers (XFELs) has catalyzed development of integrating PADs. In an XFEL, the X-ray pulse duration is typically femtoseconds long, and a new sample is needed for each X-ray pulse. Detector requirements are largely dictated by the repetition rate of the XFEL, with single-frame detectors suitable at warm-linac facilities which operate in the 100 Hz range (Philipp *et al.*, 2011*a* ▸,*b* ▸). The superconducting linac XFELs can produce bursts of pulses with pulse repetition rates of 4.5 MHz, necessitating the use of fast frame storage mechanisms (Henrich *et al.*, 2011 ▸) similar to the detector described in this paper.

## 2. Detector description   

### 2.1. Detector module: ASIC and diode   

The Keck-PAD uses a custom CMOS ASIC fabricated using TSMC 0.25 µm mixed-mode technology. The ASIC has a pixelated design with per-pixel over-glass openings and contacts to allow for the pixel-level conveyance of charge carriers from a detector diode layer. The pitch of the pixels is 150 µm in both directions with a 100% fill factor.^1^ The I/O pads are restricted to a single row along one edge of the ASIC, allowing for close tiling of multiple ASICs on the remaining three sides. Table 1 ▸ is provided for quick reference for ASIC and detector specifications. The diode detection layer is made from 500 µm-thick high-resistivity n-doped silicon and is used to absorb incident X-rays, converting the absorbed energy to charge carriers in the silicon. The diode is pixelated on one side and mates to the CMOS ASIC with p++ doping, pixel-level aluminium pads and over-glass openings. The front side of the diode, the side that does not mate to the ASIC, is not pixellated and is n-doped with a thin aluminium contact (much less than 1 µm) that is used to globally apply a reverse bias across the thickness of the diode. With this structure, holes are collected by the pixel inputs through bump-bonds. In operation, a reverse bias of approximately 200 V ensures over-depletion of the bulk silicon, and rapid collection of the charge (under normal circumstances within tens of nanoseconds). The diode is fabricated by SINTEF (Trondheim, Norway). Post-fabrication processing of the diode and ASIC wafers are performed by RTI International (NC, USA), involving pixel-level mating using Pb-Sn eutectic solder bumps. Post-processing includes deposition of under-bump metallization, additional polymer passivation, and solder bumps on the ASIC; and deposition of Ni-Au metal and additional polymer passivation on the diode to allow good adherence of bumps in the final mated hybrid module.

**Figure 1:**
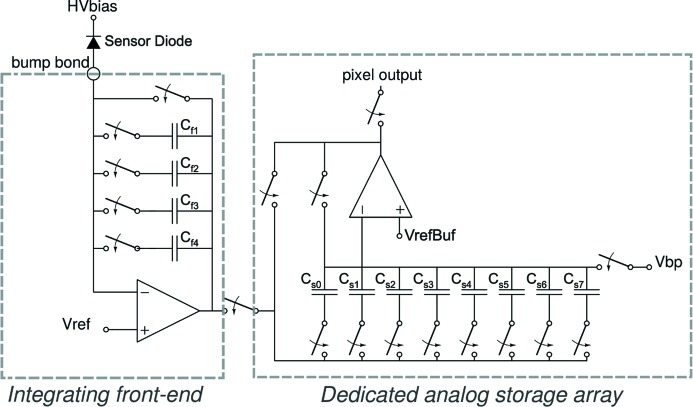
The pixel comprises a front-end integrating amplifier stage with four selectable integration capacitors and a reset switch, an array of sample-and-hold capacitors that are used to store analog values, and a readout amplifier stage. The sample-and-hold capacitors, *C*_s0-s7_, are uniformly 300 fF and are addressed in parallel across the detector so that each capacitor value represents a pixel in a stored frame. The feedback capacitors, *C*_f1-f4_, have approximate values of 300 fF, 466 fF, 500 fF and 700 fF. An externally supplied reference voltage, *V*_ref_, defines the virtual voltage for the pixel input attached to the bump-bond. *V*_refBuf_ and *V*_bp_ are externally supplied reference voltages. All switches are fast CMOS switches designed to reduce charge injection (Koerner & Gruner, 2011 ▸).

High-speed framing requires a front-end amplifier that can slew fast and settle quickly so the output can be sampled before the next integration period. This is accomplished with a differential class AB amplifier, described in detail previously (Koerner *et al.*, 2009 ▸). The key feature of this amplifier is the ability to provide a high slew current in response to transient signals without a significant increase in quiescent current. The high-level pixel schematic is shown in Fig. 1 ▸. The AB amplifier is used as an integrating amplifier with four selectable integration capacitors. The capacitors can be selected in any combination to yield 15 different gain settings, with the feedback capacitance settable from 300 fF to 1966 fF. The output of the integrating front-end amplifier is sampled by eight individually selectable storage capacitors that can be controlled at high speeds, allowing for the capture of sequential frames. The values stored on the capacitors are then read out at a slower frame rate. The present electronics can read out at up to 1.16 kHz. For simplicity in this paper, we paraphrase and say the detector can be read out at a kHz frame rate.

The four front-end integration capacitors, 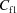 to 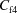, can also be used as cyclically addressed integration buffers to improve the signal-to-noise ratio for weak cyclically repetitive X-ray signals. This mode of operation is described by Koerner & Gruner (2011 ▸) and Koerner (2010 ▸). The implementation of this feature in the pixel is the same as in the prototype pixel described in these references but has not been explicitly tested in the present ASIC, so it will not be further discussed here.

**Figure 2:**
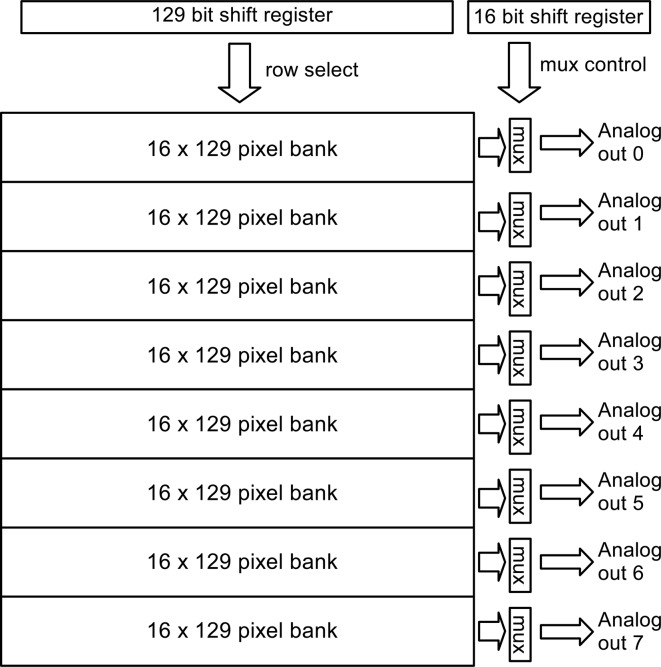
Each ASIC is divided into eight banks of pixels. Each bank is 129 × 16 pixels with a dedicated analog output for each bank. Rows are addressed in parallel across all banks with bank-wise analog multiplexers at the top of the columns.

The detector described is a scaled-up version of a 16 × 16 pixel detector covered in a previous report (Koerner & Gruner, 2011 ▸); see that paper for additional information of pixel design and test of linearity and radiation hardness. The present chip has 128 × 129 pixels per monolithic CMOS die. The 129th row was left un-bonded to the detector diode layer to allow for frame-by-frame offset correction if needed.^2^ A full-scale chip architecture, emphasizing the addressing scheme, is shown in Fig. 2 ▸. After a high-speed imaging sequence, the analog values stored in the pixels are read out using the diagrammed shift-register based addressing scheme. A large, 129-stage, shift-register passes an addressing bit that is fanned out as a select signal to all pixels in a row. Analog multiplexers define eight banks of pixels, each being 16 pixel-columns wide. The bank multiplexers are controlled in parallel with a single 16-bit shift-register that cycles though the columns for each row selected. Each analog multiplexer produces a buffered output that is routed to a wire bonding pad for digitization off-chip. In the current prototype, off-chip digitization is performed with a 12-bit ADC.

The most straightforward mode of operation is to set the gain by choice of the desired combination of simultaneously engaged integration capacitors (from 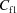 to 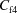). One then successively performs up to eight sequential integrations, each in response to an external trigger signal, with the voltage from each integration stored on the analog storage array capacitors 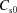 to 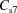. However, since each CMOS switch may be independently controlled by appropriate programming of the field-programmable gate array (FPGA), it is also possible to operate the ASIC in a mode whereby one then successively stores four additional frames, albeit with different gains, on 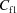 to 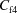. Only the former mode was used for the tests described in this paper.

### 2.2. Detector support electronics   

**Figure 3:**
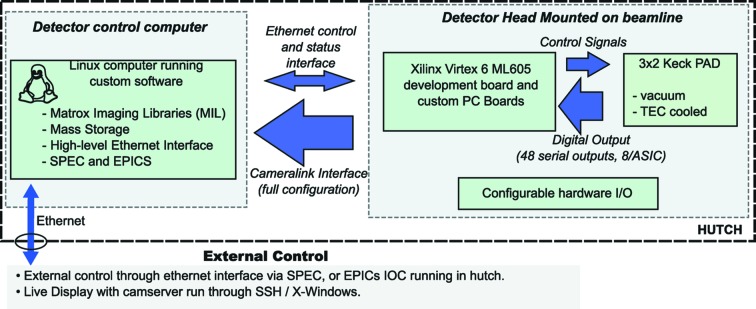
A control diagram showing the Keck-PAD system can be described with two fundamental units, the detector head and the detector control computer. The detector head has an FPGA development board, the hybrid modules, support electronics and configurable hardware I/O. The detector control computer runs *Camserver* on a Linux platform and uses the Matrox Imaging Libraries (MIL). Two basic modes of communication between the detector head and the detector control computer are used, an ethernet connection for sending control and status packets, and a *Cameralink* (full configuration) connection that is used primarily for the transfer of raw imaging data from the detector head to the detector control computer.

**Figure 4:**
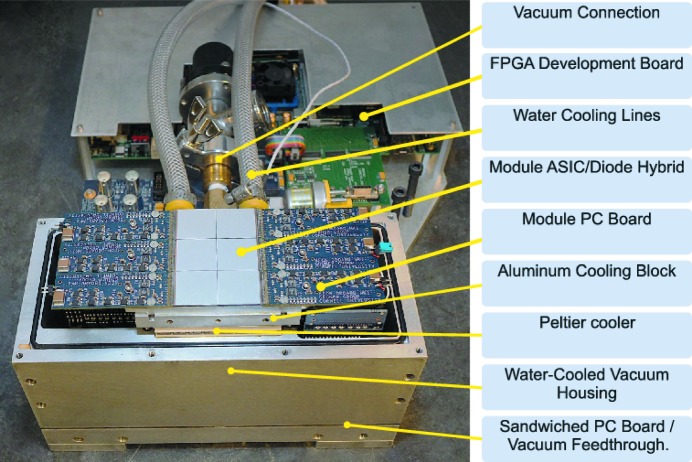
The 3 × 2 Keck-PAD array in a vacuum housing with the front window removed to show front-end electronics extending from the sides of the detector modules. During operation, the detector array and front-end electronics are held in vacuum and the temperature of the hybrid modules is actively regulated by a thermoelectric cooler to ±0.1 K in the 253–243 K range. Transfer of heat from the detector head is performed by water-cooling lines held at 283–288 K. An FPGA development board is integrated into the detector head for configurable detector control.

Detector support electronics are responsible for control of the Keck-PAD hybrid modules, synchronization of data taking with synchrotron bunches, triggering data acquisition using signals from the experiment, and the collection and storage of data. The system is diagrammed at a high level in Fig. 3 ▸ and has two main components: the detector head, mounted on the beamline, and the detector control computer. The detector head, shown in Fig. 4 ▸ with the X-ray window removed, consists of a vacuum housing that contains front-end electronics and hybrid detectors; and a FPGA development board (Xilinx, Virtex-6 ML605) that interfaces the front-end electronics *via* custom PC boards. The front-end electronics, shown in Fig. 4 ▸, consist of six identical modular units, each interfacing a single hybrid module. Each modular unit comprises a PC board that is wire-bonded to one of the six hybrid modules extending in the plane of the hybrid module, and a second board (right-angle board) that connects the daughter board to a backplane. Analog buffers for the output of the ASIC reside on the daughter board in close proximity to the hybrid module. Each daughter board also features digital potentiometers that are used for local control of detector current biases and voltages. Analog to digital conversion is performed on the right-angle board with each module producing a serial digital stream for each of the eight analog outputs. The 48 digital output streams are routed on the backplane board, which also serves as a vacuum feedthrough, and a small ‘fanout’ board to the FPGA development board. Digital signals for control and readout of the detector modules are routed in a similar fashion with digital buffers on the right-angle boards.

The Virtex-6 development board controls the detector with a highly configurable state machine that produces waveforms for a large number of possible operational modes. These modes include programmable burst modes in which integration times and frame periods are pre-programmed and triggered by a hardware signal. A subset of these modes are described in a later section. Prior to data taking, the detector status and mode is set by the FPGA board that interfaces the detector control computer through a dedicated ethernet connection. Biases, frame timing, triggering and any delays necessary for synchronizing the Keck-PAD with an experiment are set using this low-level interface. The user controls this interface through high-level commands available *via* a detector control program. The program runs with a TCP/IP socket interface and can be controlled with SPEC, EPICS or other software capable of sending and receiving strings though a TCP/IP socket.

After frames are acquired and stored in-pixel, the ASIC is read out at a frame rate of 1 kHz. The data streams are parsed and sent to the detector control computer over *Cameralink* (full configuration). A dedicated *Cameralink* card controlled by the Matrox Imaging Libraries (MIL) is used to provide a controlled data flow into a dedicated memory space buffer. The contents of this buffer are then written to local mass storage. MIL also provides a rich set of library functions that include access to a live display during detector readout. The live display provides immediate user feedback during alignment and data acquisition.

### 2.3. Operational modes   

**Figure 5:**
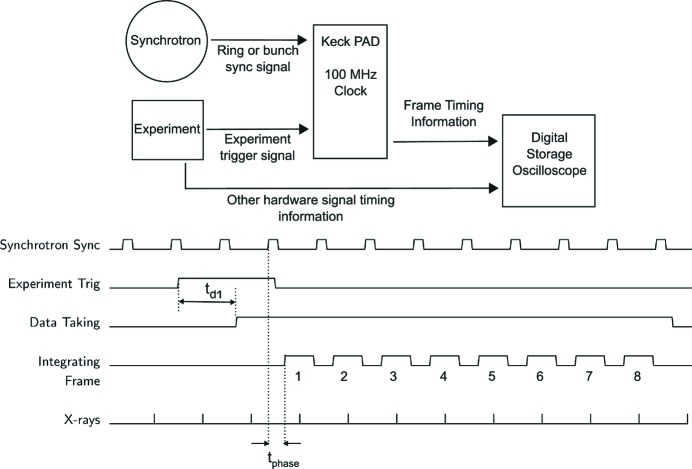
A minimal timing diagram for an experiment using the Keck-PAD and single-bunch imaging is shown. Imaging with the Keck-PAD detector needs signals from both the synchrotron and the experimental setup to capture relevant data. A synchrotron sync signal provides information about the arrival time of X-ray pulses. In practice, a phase correction, *t*_phase_, needs to be applied to capture bunches. At least one experimental trigger is also required, indicating that the dynamic process under investigation has been initiated, and that after some delay, *t*_d1_, imaging should proceed with the next bunch.

The configurable state-machine in the Virtex-6 development board gives the Keck-PAD an abundance of operational modes. A typical simplified experimental setup is shown in Fig. 5 ▸. Time-resolved measurements that take advantage of bunch structure commonly need to synchronize imaging with the experiment as well as the arrival of X-rays produced by a single, or small integer number of, bunch(es). Hardware triggering inputs to the detector are essential for low latency and repeatability. Synchronization signals at synchrotrons often only supply the bunch pattern (*e.g.* pulse arrival frequency or storage ring period) with an arbitrary phase offset. The phase offset is handled by a variable delay in the FPGA that adds time between the synchrotron trigger and the waveforms associated with framing the detector. The FPGA clock introduces a 10 ns uncertainty at the start of each data sequence. The integration window for each frame must be long enough to encompass this jitter as well as the collection time for charges produced in the diode, typically of the order of tens of nanoseconds.

A typical experiment might proceed as follows (Fig. 5 ▸). A pump event is triggered or an event is detected in the experiment. This sends a TTL trigger to the detector and the FPGA takes this as a pre-trigger indicating that after some predefined delay, 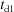, determined by the experimental needs, the next positive rising edge of the synchrotron sync signal will start a data taking sequence. When the synchrotron sync signal is received, there is some phase delay, 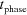, before framing begins. As framing proceeds, an output signal from the detector head indicates the framing of the detector. This information can be recorded by a digital storage oscilloscope, along with other relevant signals, to record the precise collection time of images.

## 3. Detector performance and characterization   

### 3.1. Gain and noise   

**Figure 6:**
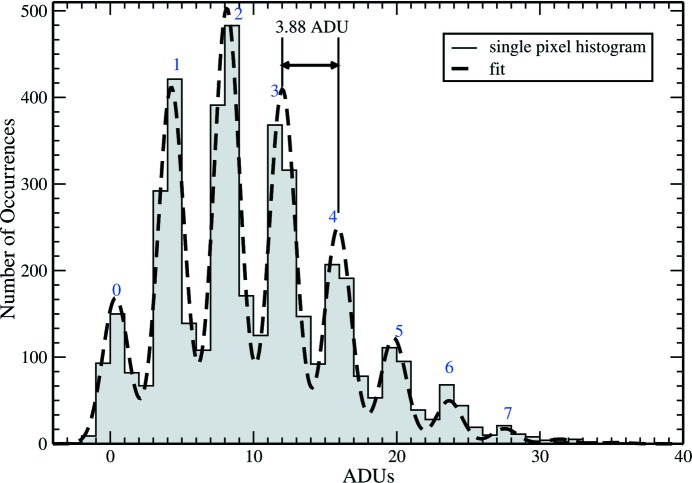
Histogram of pixel response for low-intensity illumination by 17.5 keV X-rays. Discrete peaks for 0–7 photons per pixel are indicated. Data taken at highest gain yields a fit of 3.88 ADU per X-ray (at 17.5 keV) and 1.3 ADU noise.

The gain and noise of an integrating pixel array detector can be directly measured by illuminating the detector with a monochromatic X-ray source and taking frames in the low-fluence regime. Each X-ray photon results in a well defined amount of charge with one electron–hole pair per 3.65 eV of absorbed X-ray energy. The total charge recorded increments in discrete steps for each X-ray photon. Histogramming the pixel response over a large number of frames yields a discrete photon spectra. Fig. 6 ▸ shows a characteristic spectrum for a pixel illuminated with a low flux of 17.5 keV X-rays. To obtain the spectrum, a molybdenum X-ray tube was run at 24 kV, 0.350 mA. The beam was filtered with a 200 µm-thick zirconium foil to reduce the fraction of Bremstrallung radiation relative to the 17.5 keV characteristic line. X-ray pinholes 75 µm in diameter were centered on selected pixels to mask pixel border regions from X-rays and ensure selective illumination of the central portion of the pixel, reducing the number of X-rays producing charge shared between adjacent pixels. Three thousand frames were taken at the highest gain setting (with 300 fF feedback capacitance) with an average of 3 photons per frame per masked pixel. The spectrum can be fit to a series of Gaussian peaks of uniform width and spacing, with the magnitudes corresponding to the expected Poisson distribution with the average number of photons being a freely fit parameter. The result of the fit shows peak spacing of 3.88 ADU, corresponding to a single 17.5 keV photon, yielding a gain of 1 ADU per 4.51 keV of absorbed X-ray energy. The width of the peaks indicates a standard deviation of 1.3 ADU, corresponding to an equivalent noise of 1600 electrons or 5.9 keV. Given the 300 fF feedback capacitor used, this also corresponds to 860 µV total noise on the pixel output voltage.

Pixel saturation, as measured with a laboratory X-ray source, occurs at about 2200 ADU. This corresponds to a 1.45 V swing in the output of the integrating front-end and a full well of approximately 1200 8 keV X-rays in high gain. With all feedback capacitors, *C*_f1-f4_, switched on, this corresponds to a low-gain saturation signal of 8000 8 keV X-rays. The noise of 0.7 8 keV X-rays for high gain and the full well of 8000 8 keV X-rays together give a measurement of the dynamic range without specific regard for dynamic detector performance (*i.e.* with high-speed imaging the charge migration times across the diode, the slew rate of the front-end pixel amplifier and the temporal structure of the source need to be considered). The slewing capability of the front-end has been covered elsewhere (Koerner *et al.*, 2009 ▸).

### 3.2. Dynamic testing   

**Figure 7:**
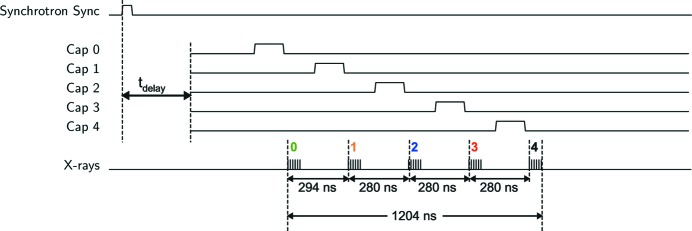
Testing the isolation of bunch trains at CHESS was carried out by synchronizing successive front-end integration times with a synchrotron synchronization signal and imposing a variable delay, *t*_delay_. The output of the integration stage was then captured on sampling capacitors (Cap 0–Cap 4). A ‘high’ signal for each sampling capacitor represents the integration window of the respective capacitor in the conceptual timing diagram. By successively advancing *t*_delay_, a sequence of integrations can be phased with bunch trains, mapping bunch train profiles with a time resolution limited only by the intrinsic time resolution of the detector (see Fig. 8 ▸).

The dynamic performance of the detector was measured directly using the positron bunch structure circulating in the Cornell Electron Storage Ring (CESR), at the F3 beamline at the Cornell High Energy Synchrotron Source (CHESS). The positron bunch structure at CESR consists of five groups of bunches (‘bunch trains’). Each bunch train contains six bunches and has a total duration of 70 ns. The ring period is 2.563 µs, and the bunch trains occupy a 1.204 µs portion of the total period. Fig. 7 ▸ shows the spacing between bunch trains: a nearly regular period of 280 ns with the spacing between the first and second trains deviating from this with an additional 14 ns (*i.e.* the time between the arrivals of the two leading bunch trains is 294 ns, not 280 ns). An additional feature of the bunch fill pattern is that the middle bunch train, labeled as bunch number 2 in Fig. 7 ▸, has a leading bunch that is under-filled such that this bunch produces fewer X-rays (proportional to the number of positrons) than the other bunches.

**Figure 8:**
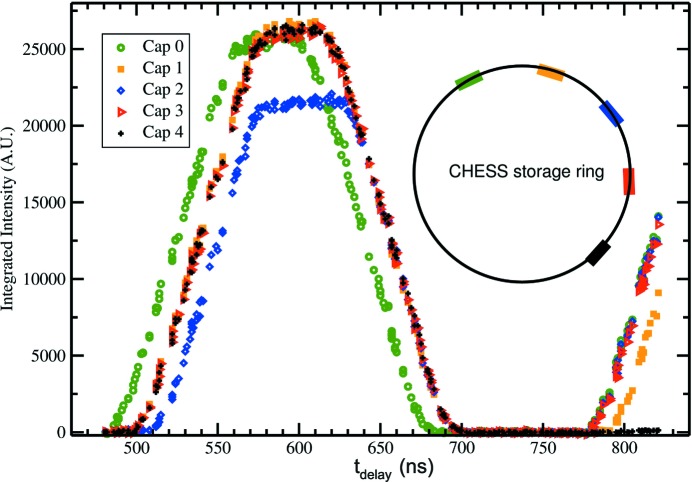
Resolving CHESS bunch train patterns was demonstrated by synchronizing the framing of the detector with the ring, setting the frame times for successive integration periods to capture different bunches, and phasing 140 ns integration windows with the ring synchronization signal by increasing the delay signal, *t*_delay_. Shown are the output from the first five capacitors as a function of phasing (delay) time. Each capacitor output is sampled signal from different bunch trains.

The bunch structure was profiled by taking eight successive images with 140 ns integration time and a 280 ns frame period. The delay between the synchrotron bunch clock and the integration period was advanced for each set of eight frames, sweeping the 8 keV X-rays pulses through the integration window of each frame progressively. The illuminated pixels (*e.g.* 10 × 10 pixels) are summed for each frame and plotted in Fig. 8 ▸.

Each of the bunch trains is resolved. In addition, the characteristics of the fill pattern described above are seen. The first bunch train, captured by Cap 0, arrives earlier than one would expect with a uniform 280 ns period. The bunch captured with Cap 2 is the under-filled bunch train. The magnitude of the bunch is less than the others by the expected amount; the plateau is 5/6 of the height of the others. In addition, the middle bunch also produced a wider plateau because the bunch train is effectively shorter in duration and is convolved with the detector integration time. As the delay is increased, each of the capacitors start to detect subsequent bunches, and, again, the bunch spacings are consistent with the CESR fill pattern. No modulation is observed at the 14 ns spacing of the bunches within the train and none is expected because the charge collection from the diode takes tens of nanoseconds. The X-ray energy used for this measurement was 8 keV.

This bunch train isolation at CHESS was used to measure detector performance because this is what was available. The bunch pattern is not, however, ideal for testing the limits of detector frame speed. As part of a scientific collaboration, high-speed framing and single-bunch isolation has recently been verified at the APS with a period of 153 ns (data not shown). Since beam time was not dedicated to detector testing, the kind of detailed analysis shown for the CHESS bunch trains was not performed.

### 3.3. Radiation-induced offset during readout   

**Figure 9:**
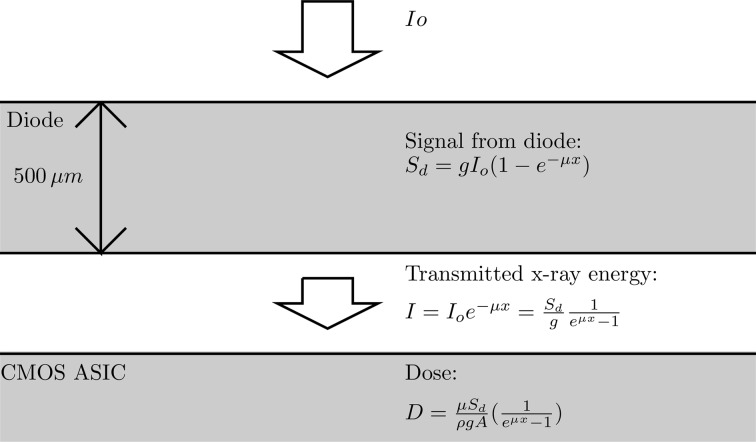
The approximate dose per bunch train was calculated as shown above. 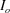 is the incident X-ray energy, *g* is the gain in units of ADU keV^−1^, 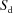 is the difference in output between illuminated and unilluminated integrations, μ is the mass absorption coefficient (taken here also to be roughly equivalent to the mass attenuation coefficient), *A* is the area of the pixel, and ρ is the density of silicon. This simplified calculation neglects absorption by, for example, solder bump-bonds.

**Figure 10:**
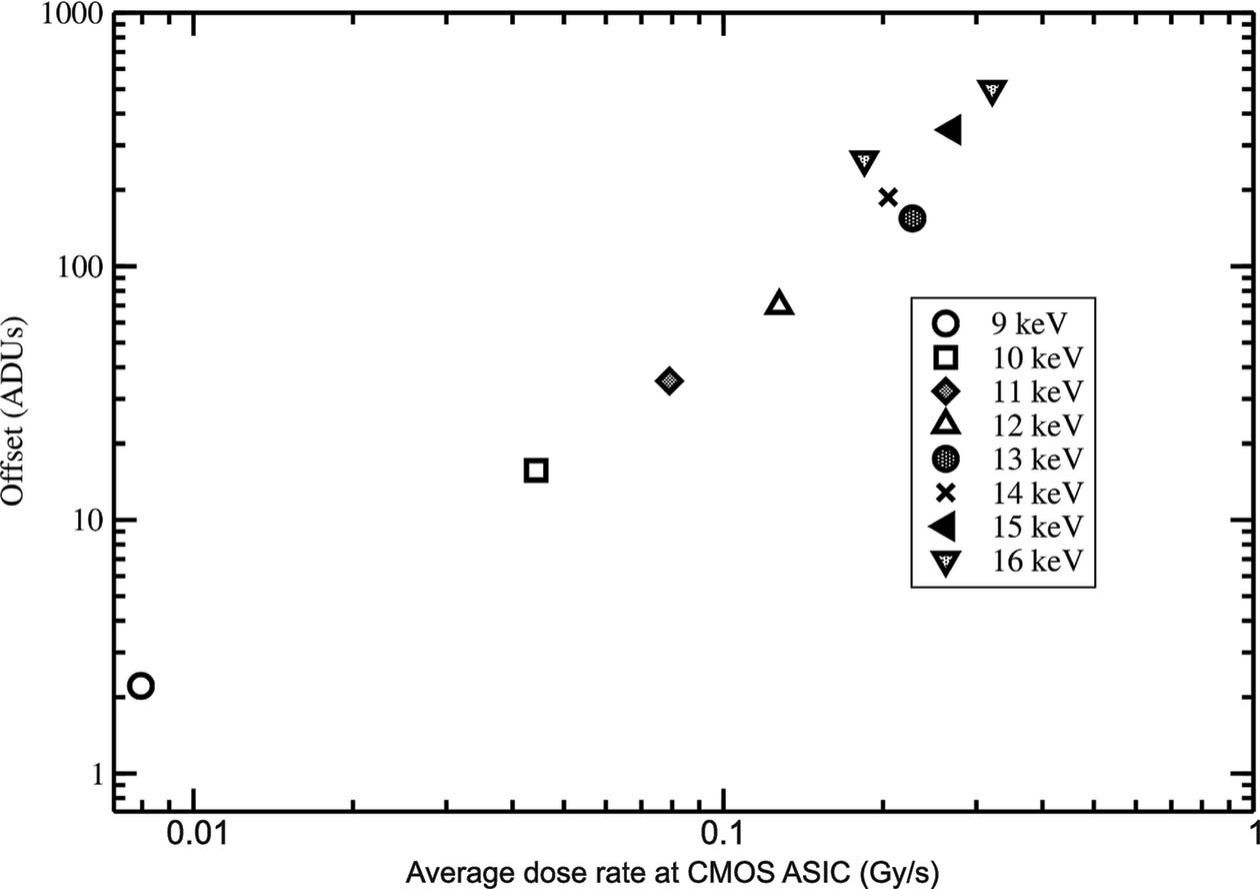
When detector frames are read out in the presence of intense X-ray illumination, an offset is imposed on the pixel output. The offset is a strong function of the dose rate received by the underlying CMOS ASIC during readout. This plot suggests a power law relationship between offset and dose rate (Gy s^−1^) of the CMOS ASIC. If the CMOS ASIC is not illuminated during readout (*e.g.* the source is shuttered), no offset is observed.

Testing with intense high-energy X-rays and short integration times showed a negative offset which was induced by direct illumination of pixels during readout. For example, with 16 keV X-rays incident an integration window was chosen in which no bunch train arrived, but when the detector was illuminated by intense radiation during readout an inverse image of the beam was apparent. If a mechanical X-ray shutter was closed prior to readout, no offset was observed. Similarly, if the integration sequence captured signal from a bunch train, but was illuminated during readout, the negative offset was still present and added to the positive signal. If no illumination was present during readout (shutter closed), the offset was not present. The effect was not present at 8 keV. At 8 keV, the attenuation length in silicon is ∼70 µm, and nearly all the X-rays are absorbed in the 500 µm sensor. With higher-energy X-rays, the offset becomes more apparent as more X-rays penetrate to the underlying ASIC, as was the case at 16 keV. Data were collected at CHESS using a range of X-ray energies and incident fluxes. The dose rate to the ASIC, which underlies the sensor, is computed as shown in Fig. 9 ▸. Fig. 10 ▸ plots the offset *versus* the dose rate at the ASIC for a variety of energies and fluxes. Because the offset is only present when the detector is illuminated during readout and correlates with the dose rate of the ASIC, the data strongly suggest that there is an offset induced in the pixel output amplifier. This is a plausible explanation because the pixel output is buffered to the chip edge with a single-ended amplifier and signal transmission assumes a common analog ground reference level. A penetrating X-ray beam could induce a change in the local value of ground, which could result in the observed offset.

In practice, the implications of this offset are mitigated simply by delaying readout of the frames until mechanical shutters are closed for the 1–8 ms readout of frames. Our practice is to include fast mechanical shutters in experiments to limit radiation exposure of the detector between data acquisition periods. In this way, readout delay implemented by the FPGA after acquisition and before readout completely removes the offset effect. The offset can also be eliminated in future versions of the Keck-PAD by redesign of the output amplifier. No discernible effect appears to persist in the behavior of the amplifier.

## 4. Experimental use   

The utility of the Keck-PAD has been demonstrated by using it to monitor and record changes in the diffraction pattern of polycrystalline magnesium alloys subjected to high-speed dynamic loading in a Kolsky-bar experiment at CHESS beamline G3 (Lambert *et al.*, 2014 ▸). This type of experiment has numerous applications in material science because it allows for the direct observation of intermediate states that are otherwise difficult to observe directly.

In this experiment, a gas gun was used to fire a projectile onto the end of a long aluminum bar. The resulting strain wave propagated down the bar at the speed of sound in steel and compressed a metal sample. The gas gun was fired asynchronously with CESR and provided a good test of the detector triggering systems. The detector was triggered with strain gauges in the transmitter bar and began integration phased to the synchrotron bunch structure.

**Figure 11:**
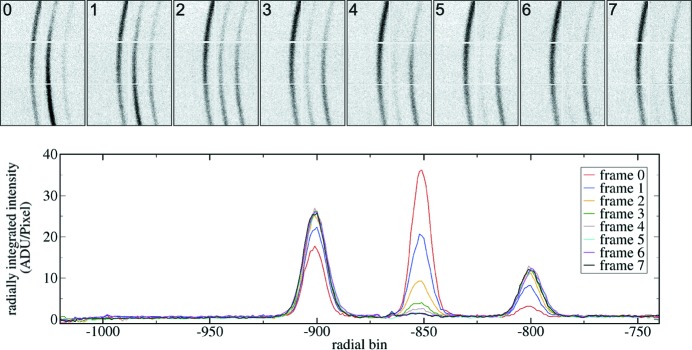
A sequence of eight diffraction images from a pure magnesium sample subjected to dynamic compression collected with the Keck-PAD is shown above along with the per-frame signals integrated over a fixed azimuthal angle. Each frame was acquired using a 5 µs integration time that captured ten bunch trains at CHESS beamline G3. The period between frames was 7.5 µs. The data shown are uncorrected with only an average dark signal subtracted from the data frames.

**Figure 12:**
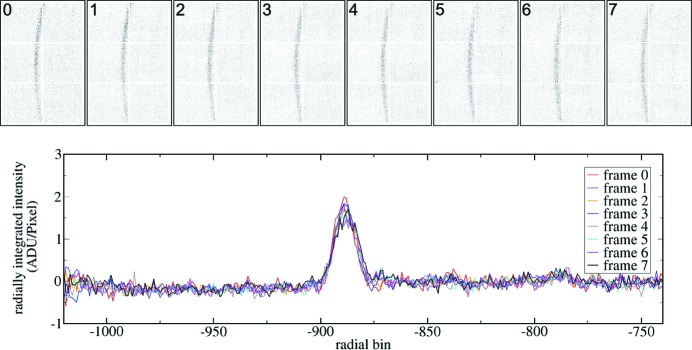
A sequence of eight diffraction images from a magnesium alloy sample subjected to dynamic compression collected with the Keck-PAD is shown above along with the per-frame signals integrated over a fixed azimuthal angle. Each frame was acquired using a 180 ns integration time that captured one bunch train at CHESS beamline G3. The period between frames was 10 µs. The signal gain was 2.2 ADU per X-ray. A baseline correction has been performed on each detector tile (*i.e.* a constant value has been subtracted). In addition, four pixels in frame 4 had bit errors (an offset of 1024 ADU) that were corrected. This does not occur with later FPGA implementations.

Readout of collected frames from the ASIC was performed after closing a shutter to eliminate the effects of offsets described in the previous section. The energy of the X-rays was 10 keV. Figs. 11 ▸ and 12 ▸ show examples of frame sequences collected using the Keck-PAD. Fig. 11 ▸ is typical of the data collected by the Keck-PAD during the experiment, and shows the changing intensities of the 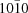, 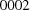 and 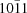 lines of a magnesium alloy sample during dynamic loading. The integration period for this data was 5 µs, allowing for the capture of ten CHESS bunch pulses. Fig. 12 ▸ shows a sequence of single-bunch images with a 10 µs frame period. Note that intensity within these frames is 1/10 of that in Fig. 11 ▸, but the time resolution is increased by a factor of 70.

## 5. Conclusion   

The full-sized Keck-PAD represents the latest generation of high-speed imagers developed in our group. The 3 × 2 tiling of 128 × 129 pixel ASICs [similar to the tiling of the MMPAD (Tate *et al.*, 2013 ▸)] represents a major advance in the sub-microsecond imaging using synchrotron sources. The ability to image using single bunches and rapidly acquire temporal snapshots of dynamic systems takes full advantage of the inherent pulsed nature of synchrotron sources.

Single-bunch train isolation has been demonstrated at CHESS. Very recently, experiments have been carried out at the APS where bunch widths are ∼30 ps with a period of 153 ns. The detector performed well. It is hoped and expected that easily accessing these time scales with fast framing detectors will open new avenues of study. Of particular interest are those experiments that may not be repeatable in detail or in which sample supply is limited so that single-shot X-ray imagers are unable to unlock a meaningful progression of states. Even for repeatable pump–probe type experiments that can theoretically be observed in detail using fast shutters and slower detectors, the gain of efficiency offered by quick capture of successive images is significant.

An updated ASIC has been fabricated and is undergoing testing. The ASIC is designed to provide improved performance by offering more dedicated storage capacitors (27 storage capacitors in each pixel) and improved single-photon signal-to-noise ratios that can, under low-fluence conditions, lead to the ability to threshold detector output and reduce the effects of systematic detector artifacts (Philipp *et al.*, 2011*b* ▸). Since low-fluence data often accompanies high-speed imaging, good signal-to-noise ratios for single photons are imperative for maximizing the scientific utility of the detector.

## Figures and Tables

**Table 1:** Selected specifications of Keck-PAD

Pixel size	150 µm × 150 µm
Tile size	128 × 128 pixels
Tiled array	3 × 2 tiles
Noise (V)	860 µV (high gain)
Noise (X-ray equivalent)	0.7 8 keV X-ray (high gain)
Digital gain	1.77 ADU / 8 keV X-ray
Full well	1200 8 keV X-rays (high gain)
	8000 8 keV X-rays (low gain)
Frames stored	Up to 8
Maximum stored in-pixel frame rate	∼10 MHz (6.5 MHz X-ray imaging verified)
Read time	<1 ms per stored frame

## Footnotes

1 The fill factor is defined as the fraction of imaging area that is sensitive to X-rays.

2 Initial testing of this technique for small offset corrections has not demonstrated this to be an effective technique. More investigation is needed.
